# The periovulatory endocrine milieu affects the uterine redox environment in beef cows

**DOI:** 10.1186/s12958-015-0036-x

**Published:** 2015-05-10

**Authors:** Roney S Ramos, Milena L Oliveira, Aryele P Izaguirry, Laura M Vargas, Melina B Soares, Fernando S Mesquita, Francielli W Santos, Mario Binelli

**Affiliations:** Department of Animal Reproduction, School of Veterinary Medicine and Animal Science, University of São Paulo, Pirassununga, SP 13635-900 Brazil; Laboratory of Reproductive Biotechnology (Biotech), Federal University of Pampa, Uruguaiana, Brazil; School of Veterinary Medicine, Federal University of Pampa, Uruguaiana, Brazil

**Keywords:** Estradiol, Progesterone, Endometrium, Oxidative stress, Cattle

## Abstract

**Background:**

In cattle, recent studies have shown positive associations between pre-ovulatory concentrations of estradiol (E2), progesterone (P4) at early diestrus and fertility. However, information on cellular and molecular mechanisms through which sex steroids regulate uterine function to support early pregnancy is lacking. Based on endometrial transcriptome data, objective was to compare function of the redox system in the bovine uterus in response to different periovulatory endocrine milieus.

**Methods:**

We employed an animal model to control growth of the pre-ovulatory follicle and subsequent corpus luteum (CL). The large follicle-large CL group (LF-LCL, N = 42) presented greater levels of E2 on the day of GnRH treatment (D0; 2.94 vs. 1.27 pg/mL; P = 0.0007) and P4 at slaughter on D7 (3.71 vs. 2.62 ng/mL, P = 0.01), compared with the small follicle-small CL group (SF-SCL, N = 41). Endometrium and uterine washings (N = 9, per group) were collected for analyses of variables associated with the uterine redox system.

**Results:**

The SF-SCL group had lower endometrial catalase (0.5 vs. 0.79 U/mg protein, P < 0.001) and glutathione peroxidase (GPx; 2.0 vs. 2.43 nmol β-nicotinamide adenine dinucleotide phosphate reduced/min/mg protein, P = 0.04) activity, as well as higher lipid peroxidation (28.5 vs. 17.43 nmol malondialdehyde/mg of protein, P < 0.001) and superoxide dismutase (SOD) activity (44.77 vs. 37.76 U; P = 0.04). There were no differences in the endometrial reactive species (RS) or glutathione (GSH) concentrations between the groups. The uterine washing samples showed no differences in the concentrations of RS or GSH or in total SOD activity (P > 0.1). Additionally, catalase, *GPx4*, *SOD1* and *SOD2* gene expression was lower in the SF-SCL group than in the LF-LCL group.

**Conclusions:**

We concluded that the intrauterine environment of cows from the LF-LCL group exhibited higher antioxidant activity than that of the cows from the SF-SCL group. We speculate that uterine receptivity and fertility are associated with an optimal redox environment, such as that present in the animals in the LF-LCL group.

## Background

Current research has indicated that a larger pre-ovulatory follicle [1-3] and a longer duration of proestrus [3,4], as well as higher concentrations of pre-ovulatory estradiol (E2) [5] and post-ovulatory progesterone (P4) have beneficial effects on the fertility of beef cattle [1,6]. However, the mechanisms by which the timing and prominence of these hormones around ovulation act to improve fertility remain unknown. Our overarching hypothesis was that the action of reproductive hormones modulates oviductal and uterine function to support preimplantational embryo development.

There is evidence of a direct association between changes in the production of E2, P4, and their respective receptors and changes in the abundance of transcripts, synthesis, and the secretion of proteins in the oviduct and the endometrium [7-9]. Recent studies have shown a positive association between pre-ovulatory concentrations of E2 and the duration of proestrus regarding the uterine environment and fertility [4,10]. Moreover, other studies have shown that P4 supplementation in early diestrus altered global gene expression in the endometrium of beef cows [11,12]. Comprehension of the mechanism whereby these factors can affect the fertility of beef cattle is important, and additional knowledge about it could be used for the development of novel strategies to improve the fertility of beef cattle.

Control of the redox environment by sex steroids is a critical process that may be involved in uterine receptivity that has never been studied during pre-implantation in cattle.

The redox environment is a reflection of the state of different redox couples (oxidized/reduced molecules) that are in balance between the products of the reduction potential and reducing capacity where these couples are responsive to changes in a reducing/oxidizing environment [13]. Regulation of the reducing capacity, provided mainly by antioxidant enzymes, such as superoxide dismutase (SOD), catalase (CAT), and glutathione peroxidase (GPx), is important because the metabolites of the oxidative process participate in several cellular processes, such as protein phosphorylation, phospholipid hydrolysis, activation of transcription factors, and inhibition of phosphatases [14], in addition to their known action in the damage caused by oxidative stress. Role of reactive oxygen species and oxidative stress in female reproduction has been well reviewed [15,16]. Studies with E2 and P4 have shown that these ovarian steroids regulate GPx activity [17,18] and glutathione reductase levels in rats [19], as well as SOD1, CAT and GPx activities in sheep [20]. Furthermore, our recent data indicated that the oxidation-reduction process (GO:0055114) was a functional gene category that was enriched in the endometrium of animals treated to ovulate large follicles (Mesquita FS, Ramos RS, Pugliesi G, Andrade SCS, Oliveira ML, Gonella-Diaza AM, et al.: The receptive endometrial transcriptomic signature indicates an earlier shift from proliferation to metabolism at early diestrus in the cow, submitted). Thus, we hypothesized that fluctuation of sex steroid concentrations around ovulation could alter the redox environment and regulate the quality of the uterine environment in beef cows.

The present study aimed to determine whether the reactive species (RS) and other components of the redox system were regulated by the periovulatory endocrine milieu in the uterus of beef cows during early diestrus.

## Methods

### Animal handling and sampling procedures

The study was conducted at the University of São Paulo in Pirassununga, Brazil, and the animal procedures were approved by the ethics committee of the University of São Paulo (protocol No. 2287/2011).

**Figure 1:**
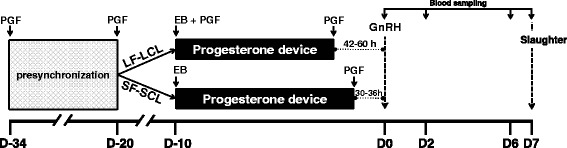
Synchronization protocol. Cows were pre-synchronized via two injections of prostaglandin F2α (PGF) administered 14 days apart, starting on protocol Day −34 (D–34). On D–10, the cows received a progesterone-releasing device (P4 device) (Sincrogest; Ourofino Saúde Animal, Cravinhos, SP, Brazil) and an injection of 2 mg of estradiol benzoate (EB; Sincrodiol; Ourofino Saúde Animal). Also, on D–10, the cows were prearranged between large follicle-large corpus luteum group (LF-LCL) or small follicle-small corpus luteum group (SF-SCL) and only cows of LF-LCL received an injection of PGF (0.5 mg of sodium cloprostenol; Sincrocio; Ourofino Saúde Animal). Between D–1.75 and D–2.5 the P4 device was removed from cows of LF-LCL. On the cows of SF-SCL the P4 device was removed between D–1.25 and D–1.5. All the animals received an injection of PGF in the same time of P4 device withdrawal and a second PGF injection 6 h later. On D0, in both groups, the ovulation was induced with an injection of gonadotropin-releasing hormone (GnRH; 0.01 mg of buserelin acetate; Sincroforte; Ourofino Saúde Animal). Blood sampling was conducted on D0, D2, D6 and D7. The cows were slaughtered on D7, which was the end point for endometrial tissue collection.

The samples (endometrium and uterine washings) used in the present study were the same used in previous studies [21,22] although analytical endpoints were different on those studies than the present study. Briefly, 83 Nelore cows (*Bos indicus*) at random estrous cycle stages were pre-synchronized with two injections of prostaglandin F2α (PGF; 0.5 mg sodium cloprostenol; Sincrocio®, Ourofino Saúde Animal, Cravinhos, Brazil) with a 14-day interval between doses (first PGF: D–34; second PGF: D–20). On D–10, the cows received a progesterone-releasing device (P4 device) (Sincrogest; Ourofino Saúde Animal) and an injection of 2 mg of estradiol benzoate (EB; Sincrodiol; Ourofino Saúde Animal). Also, on D–10, the cows were assigned to either the large follicle-large corpus luteum group (LF-LCL; n = 42) or the small follicle-small corpus luteum group (SF-SCL; n = 41). On D-10, cows on the LF-LCL received an injection of PGF and cows on the SF-SCL received nothing. Between D–1.75 and D–2.5 (42 to 60 h prior D0) the P4 device was removed of cows of LF-LCL. On the cows of SF-SCL the P4 device was removed between D–1.25 and D–1.5 (30 to 36 h prior D0). All animals received an injection of PGF simultaneously to P4 device withdrawal and a second PGF injection 6 h later. On D0, in both groups, ovulation was induced with an injection of gonadotropin-releasing hormone (GnRH; 0.01 mg of buserelin acetate; Sincroforte; Ourofino Saúde Animal). Blood sampling was conducted on D0, D2, D6 and D7 (Table 1 and Figure 1). Blood samples were collected using jugular venipuncture with tubes containing EDTA (BD, São Paulo, SP, Brazil) and transported on ice to the laboratory. Plasma was separated by centrifugation (within 2 hours after the collection) at 4°C, 1500 × g for 30 min (Sorvall®, RC3B Plus), and stored at −20°C. Cows were slaughtered on D7, which was the end point for endometrial tissue collection (Table 1 and Figure 1).

**Table 1:** **Experimental design**

**Procedure**	**Daytime**	
	**SF–SCL**	**LF–LCL**
*First PGF of pre-synchronization*	D–34	D–34
*Second PGF of pre-synchronization*	D–20	D–20
*P4 device insertion + EB injection*	D–10	D–10
PGF*	NO**	D–10
P4 device withdrawal + PGF***	D–1.25 to D–1.5	D–1.75 to D–2.5
*GnRH injection*	D0	D0
*Blood sampling*	D0, D2, D6 and D7	D0, D2, D6 and D7
*Slaughter*	D7	D7

*Abbreviations*: *PGF* prostaglandin F2α (sodium cloprostenol; Sincrocio®, Ourofino Saúde Animal, Cravinhos, Brazil), *P4 device* progesterone-releasing device (Sincrogest; Ourofino Saúde Animal, Cravinhos, SP, Brazil), *EB* Estradiol benzoate (Sincrodiol®, Ourofino Saúde Animal), *GnRH* gonadotropin-releasing hormone (Sincroforte®, Ourofino Saúde Animal). *PGF given on the beginning of protocol; **Cows of SF-SCL did not receive PGF injection on D–10; ***PGF given on the end of protocol.

After the slaughter the reproductive tracts were transported on ice to the laboratory within 20 min. The uterine horns ipsilateral to the ovary containing a corpus luteum (CL) were washed with 20 mL of phosphate-buffered saline (PBS). The uterine washings (representing the extracellular content present in the uterine lumen) were centrifuged at 300 × g, for 30 min, at 4°C (Sorvall®, RC3B Plus) and the supernatant was aliquoted, frozen and stored at −80°C until analyses. The uterine washings are representative of the histotroph, a collection of secretions present in the uterine lumen that are responsible for pre-implantation embryo nutrition. After washing, the uterine horns were longitudinally incised, and the intercaruncular endometrium was dissected and stored at −80°C for subsequent analysis.

### Ovarian measurements

For *in vivo* ovarian morphology evaluation transrectal ultrasonography was performed with a B-mode and color Doppler ultrasound instrument (Mylab30 vet Gold, Esaote Healthcare, São Paulo, SP, Brazil). On D–10 all the cows were scanned and only those that had a functional CL present were assigned to one of the two experimental groups (Figure 1). After P4 device withdrawal the follicular diameters were measured daily until D0. Between D0 and D2 the evaluations of follicles were performed each 12 hours to check for ovulation. On D7 the volume of CL was calculated by applying the radius of the average diameter in the spherical volume formula: (4/3) × π × r^3^, where π is a mathematical constant, and r^3^ is the average radius elevated to the power of 3 [21]. Also, on D7 after the slaughter, the ovaries were collected and the weight of CL was measured *ex vivo*.

### Quantification of hormonal concentrations

Blood samples were collected to measure plasma concentrations of E2 during proestrus/estrus and plasma concentrations of P4 during early diestrus. Estradiol concentrations were measured using a commercial RIA kit (Double Antibody Estradiol, Siemens, Los Angeles, CA, USA), as reported previously [22]. Plasma P4 concentrations were measured using a commercial kit (coat-a-count, DPC, Siemens, Los Angeles, CA, USA), as validated previously for cattle [23]. The intra- and inter-assay CV and sensitivity for P4, respectively, were 0.3%, 7.0% and 0.076 ng/mL. For E2, the intra-assay CV and sensitivity were 1.7% and 0.13 pg/mL.

### Biochemical analyses

Subsets of 9 cows per group were selected for biochemical analyzes (please see Statistical Analyses section for details). The tissues were homogenized in cold 50 mM Tris–HCl, pH 7.4 (1/5, w/v). The homogenate was centrifuged for 10 min at 3,000 × g , and the pellet was discarded, yielding a low-speed supernatant, which was used to determine the enzyme activities and the reactive species and glutathione levels. The uterine flushing samples were used as obtained.

### Protein determination

Total protein concentrations were measured in tissue homogenates and uterine washings by the Bradford method as previously described by Bradford [24], using bovine serum albumin (BSA) in Tris–HCl, pH 7.4, as the standard.

### Glutathione peroxidase (GPx) activity

The GPx activity in the supernatant (endometrium) or uterine washings was assayed spectrophotometrically using the method developed by Wendel [25], based on the glutathione (GSH)/β-nicotinamide adenine dinucleotide phosphate reduced (NADPH)/glutathione reductase system with the dismutation of H_2_O_2_ at 340 nm. Aliquots of endometrium homogenate (50 μL) or uterine washings were added to the GSH/NADPH/glutathione reductase system, and the enzymatic reaction was initiated by the addition of H_2_O_2_ (4 mM). In this assay, the enzyme activity was indirectly measured by the NADPH decay. Hydrogen peroxide was decomposed to generate oxidised glutathione (GSSG) from GSH. GSSG was regenerated to form GSH by glutathione reductase, which was present in the assay medium, with the use of NADPH. The enzymatic activity was expressed as nanomoles of NADPH per minute per milligram of protein.

### Glutathione (GSH) levels

The levels of reduced GSH were determined fluorometrically as described by Hissin [26], using o-Phthalaldehyde (OPA) fluorophore. Samples were homogenized in 100 mM perchloric acid (HClO_4_). The homogenates were centrifuged at 3,000 × *g* for 10 min, and the supernatants were separated for GSH quantification. Supernatants (100 μL) were incubated with the same volume of OPA (0.1% in methanol) and 1.8 μL of phosphate buffer (pH 8.0) for 15 min at room temperature in the dark. The fluorescence was measured with a fluorescence spectrophotometer at an excitation wavelength of 350 nm and an emission wavelength of 420 nm. A five-points curve (2.5; 5; 10; 20 and 50 nmol of GSH) was used as a standard. The GSH levels are expressed as nanomoles (nmol) of GSH per gram of tissue.

### Reactive species (RS) levels

The RS levels were determined by a spectrofluorimetric method [27], using the 2’,7’-dihydrodichlorofluorescein diacetate (DCHF-DA) assay. Supernatant (endometrium) or uterine washings were incubated with 10 μL of DCHF-DA (1 mM) at room temperature. DCHF-DA is rapidly oxidized in the presence of RS due to its highly fluorescent derivative dichlorofluorescein (DCF). The oxidation of DCHF-DA into fluorescent dichlorofluorescein was measured for the detection of intracellular RS. The DCF fluorescence intensity emission was recorded at 520 nm (with an excitation wavelength of 480 nm) 30 min after the addition of DCHF-DA to the medium. The RS levels are expressed in fluorescence units (FU).

### Lipid peroxidation (TBARS)

An aliquot (100 μL) of the homogenized tissue (supernatant) was incubated at 95°C for 2 h with 0.8% thiobarbituric acid (TBA), acetic acid buffer (pH 3.4), and 8.1% sodium dodecyl sulfate. The thiobarbituric acid reactive species (TBARS) were spectrophotometrically determined at 535 nm, as described by Ohkawa [28]. A four-points curve (1.5; 3; 6 and 9 nmol of malondialdehyde) was used as a standard. The lipid peroxidation is expressed as nmol of malondialdehyde per milligram of protein.

### Superoxide dismutase (SOD) activity

The SOD activity was measured as previously described [29]. This method is based on the ability of SOD to inhibit the auto-oxidation of epinephrine into adrenochrome. The color reaction can be monitored at 480 nm. One enzymatic unit (1 U) is defined as the amount of enzyme necessary to inhibit the auto-oxidation rate by 50% at 26°C.

### Catalase (CAT) activity

The CAT activity in the samples was assayed spectrophotometrically as previously described by Aebi [30], and this protocol involves the monitoring of the disappearance of H_2_O_2_ in the presence of the sample at 240 nm. A supernatant aliquot (100 μL) was added to 50 mM potassium phosphate buffer, pH 7.0, and the enzymatic reaction was initiated by the addition of 105 μL H_2_O_2_ diluted (300 mM). One unit of enzyme is defined as the amount of enzyme required to detect the disappearance of H_2_O_2_. The enzymatic activity is expressed as units (U) per milligram of protein (1 U decomposes 1 μmol H_2_O_2_/min, pH 7, at 25°C).

### Transcript quantification by real-time PCR (qPCR)

Subsets of 9 cows per group were selected for transcript quantification analyzes (please see Statistical Analyses section for details). Approximately 30 mg of endometrial tissues were submitted to total RNA extraction, using the RNeasy Mini columns kit (Qiagen, Gaithersburg, MD, USA) according to the manufacturer’s instructions. To complementary DNA synthesis 1 μg total RNA was treated with DNAse I followed by reverse transcription using a High Capacity cDNA Reverse Transcription Kit (Life Technologies, Carlsbad, CA, USA). Step-One Plus (Life Technologies) with SYBR® Green Chemistry was used for the amplification reactions.

Primers were designed based on GenBank Ref-Seq mRNA sequences of target genes, using the Primer Express software, version 3.0 (Life Technologies). The specificity of the designed primers was compared by Basic Local Alignment Search Tool (BLAST; http://blast.ncbi.nlm.nih.gov). PCR products of the primers designed were submitted for electrophoresis and sequencing. Details of the primers and probes are provided in Table 2.

**Table 2:** **Characteristics of the primers used for qPCR**

**Target gene**	**GenBank ID**	**Primer sequence (5’-3’)**		**Amplicon (bp)**	**Reference**
*SOD1*	NM_174615.2	F	GTTGGAGACCTGGGCAATGT	151	Primer Express*
		R	TCCACCCTCGCCCAAGTCAT		
*SOD2*	NM_201527.2	F	CCCATGAAGCCTTTCTAATCCTG	307	[47]
		R	TTCAGAGGCGCTACTATTTCCTTC		
*SOD3*	NM_001082610.1	F	GAGAGCGAGTGTAAAGCCGT	190	PrimerQuest**
		R	CCTGGAAGAGGCACACAGAG		
*CAT*	NM_001035386.2	F	CGCGCAGAAACCTGATGTC	150	Primer Express*
		R	GGAATTCTCTCCCGGTCAAAG		
*GPX4*	NM_174770.3	F	TCACCAAGTTCCTCATTGACAAGA	150	Primer Express*
		R	TTCTCGGAACACAGGCAACA		
*PPIA*	NM_178320.2	F	GCCATGGAGCGCTTTGG	70	[48]
		R	CCACAGTCAGCAATGGTGATCT		
*ACTB*	NM_173979.3	F	GGATGAGGCTCAGAGCAAGAGA	78	[48]
		R	TCGTCCCAGTTGGTGACGAT		
*GAPDH*	NM_001034034.2	F	GCCATCAATGACCCCTTCAT	71	[48]
		R	TGCCGTGGGTGGAATCA		

*Abbreviations*: *SOD1* Superoxide dismutase 1, *SOD2* Superoxide dismutase 2, *SOD3* Superoxide dismutase 3, *CAT* Catalase, *GPX4* Glutathione peroxidase, *PPIA* peptidylprolyl isomerase A (cyclophilin A), *ACTB* actin, beta, *GAPDH* glyceraldehyde-3-phosphate dehydrogenase. ID: GenBank Identification, *Primer sequences obtained using Primer Express software, version 3.0 (Life technologies, Carlsbad, CA, USA), **Primer sequences obtained using PrimerQuestQM software (IDT Technologies, Coralville, IA, USA).

Determination of PCR efficiency and Cq (quantification cycle) values per sample were performed with LinRegPCR software (http://linregpcr.nl/) as described by Ramakers et al. [31]. For data normalization, Genorm software was used as described by Vandesompele et al. [32]. Three constitutive genes – cyclophilin (*PPIA*), glyceraldehyde-3-phosphate dehydrogenase (*GAPDH*), and beta-actin (*ACTB*) – were used as inputs. The normalization factor generated by Genorm was based on the geometric mean of the most stable genes (*GAPDH* and *ACTB*).

### Statistical analyses

The experimental model was used as a paradigm for lower (SF-SCL) or greater (LF-LCL) receptivity and fertility. Adherence to each of the paradigms was measured in each animal after joint evaluation of specific ovarian and endocrine variables, specifically, concentration of P4 at D6, P4 at D6/P4 at D2 ratio, CL size at D7, CL weight, follicle size at D-2, D-1 and D0 and pre-ovulatory follicle size. Animals within each group were ranked according to responses to each variable. The nine top ranked animals of the LF-LCL group and the lowest ranked animals of the SF-SCL group were chosen for biochemical and transcript analysis. Raw data were checked to determine the normality of the residuals by the Shapiro-Wilk test, and Levene’s test was used to check for homogeneity of variances. If necessary, data were transformed by natural logarithms or ranks. Comparisons between the groups were analyzed by one-way ANOVA using the PROC GLM procedure (SAS software, version 9.2). The P4 concentration was analyzed by split-plot ANOVA, considering the effects of group, day, and their interaction using the PROC MIXED procedure (SAS, Version 9.2; SAS Institute Inc., Cary, NC, USA). The data from the biochemical analyses were compared between the groups by one-way ANOVA (STATISTICA 4.5, StateSoft, Inc. 1993, Tulsa, OK, USA).

## Results

### Animal model

**Figure 2:**
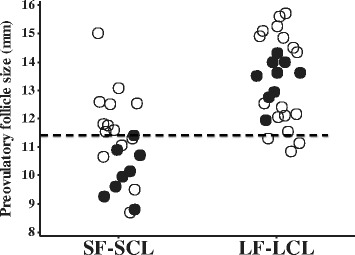
Individual preovulatory follicle (POF) sizes in each group. Cows evaluated for inclusion in the experimental groups are shown. Each dot represents a specific cow. Within each group, individual POF values were ordered from lowest to highest. The cow with the highest value received the highest score and subsequent cows received lower scores. Top ranked cows for the LF-LCL group were considered those cows with the highest scores, whereas top ranked cows for the SF-SCL group were considered those with the lowest scores. A similar procedure was conducted using additional variables, such as those associated with the CL and P4 concentrations and a compound ranking score was calculated. In both groups the top ranked cows (filled dots) were used in laboratory analysis and the remaining cows were not. The horizontal line depicts the cut-off value (11.4 mm) specifically for the POF size. Abbreviations: SF-SCL: Small follicle-small CL group; LF-LCL: Large follicle-large CL group.

The results from animal model that was used in this study was published previously by Mesquita et at. [21]. Briefly, two distinctly different groups were generated, based on ovarian morphology and function during the periovulatory period. Distribution of animals in groups according to the preovulatory follicle sizes is shown in Figure 2. The largest preovulatory follicle in the SF-SCL group was 11.4 mm, and that was the cut-off size that separated groups. Overall, the LF-LCL group was characterized by the ovulation of a follicle that was 20.2% larger, with consequent 131.5% greater E2 concentrations before ovulation, 46% larger size of the CL and 41.6% greater P4 secretory capacity during early diestrus, compared with the SF-SCL group [21]. Thus, the model was efficient to generate two groups of animals with different ovarian morphologies and endocrine conditions around ovulation. Furthermore, animals within each group were ranked according to ovarian and endocrine variables measured around ovulation and only top-ranked animals from each group were selected for analyses. Such programmed selection was meant to reduce emphasis on individual animal responses to the pharmacologic manipulations. Rather, selection aimed to define sub-groups of animals strongly displaying characteristics associated with greater (LF-LCL) or reduced (SF-SCL) pre-ovulatory follicle size. Selected animals within each group were used to analyze endometrial and uterine-flush samples regarding the redox environment.

### Biochemical analyses

The results obtained from the biochemical analyses are summarized in Table 3. Briefly, in the endometrium, the SF-SCL group showed lower CAT (0.5 vs. 0.79 U/mg protein, P < 0.001) and GPx enzymatic activity (2.0 vs. 2.43 nmol NADPH/min/mg protein, P = 0.04) than the LF-LCL group. Additionally, lipid peroxidation (28.5 vs. 17.43 nmol malondialdehyde/mg of protein, P < 0.001) and SOD activity (44.77 vs. 37.76 U, P = 0.04) were increased in the SF-SCL group, compared with the LF-LCL group. The concentrations of RS (111.12 vs. 105.4 FU, P > 0.1) and GSH (112.68 vs. 122.91 nmol GSH/g tissue, P > 0.1) were similar between the groups. The uterine flushing samples showed no differences in RS levels (25.76 vs. 37.25 FU, P > 0.1), total SOD activity (36.28 vs. 36.46 U, P > 0.1), or GSH levels (249.17 vs. 225.19 nmol GSH/mL flushing, P > 0.1) between the LF-LCL and SF-SCL groups. In this study, it was not possible to detect CAT activity and lipid peroxidation in the uterine fluid.

**Table 3:** **Results obtained from the biochemical analyses (means +/− standard error of the means)**

**Variables**	**SF-SCL**	**LF-LCL**	**P**
***Endometrium***			
*CAT activity (U/mg protein)*	0.5 +/− 0.07	0.79 +/− 0.09	<0.001
*GPx activity (nmol NADPH/min/mg protein)*	2.0 +/− 0.35	2.43 +/− 0.39	0.04
*Lipid peroxidation (nmol MDA/mg of protein)*	28.5 +/− 0.98	17.43 +/− 0.97	<0.001
*SOD activity (U)*	44.77 +/− 7.66	37.76 +/− 3.95	0.04
*Reactive species (FU)*	111.12 +/− 21.31	105.4 +/− 10.59	>0.1
*GSH levels (nmol GSH/g tissue)*	112.68 +/− 13.26	122.91 +/− 13.08	>0.1
***Uterine Flushing***			
*Reactive species (FU)*	25.76 +/− 9.81	37.25 +/− 14.98	>0.1
*SOD activity (U)*	36.28 +/− 9.64	36.46 +/− 10.3	>0.1
*GSH levels (nmol GSH/mL flushing)*	249.17 +/− 57.76	225.19 +/− 26.16	>0.1

*Abbreviations*: *SF-SCL* Small follicle-small CL group, *LF-LCL* Large follicle-large CL group, *P* P values for a one-way ANOVA, *CAT* catalase, *GPx* glutathione peroxidase, *SOD* superoxide dismutase, *GSH* reduced glutathione, *FU* fluorescence units, *U* enzymatic units, *MDA* malondialdehyde.

These results showed lower antioxidant activity (i.e., CAT and GPx activities) in the SF-SCL group in comparison to the LF-LCL.

### Abundance of transcripts

**Figure 3:**
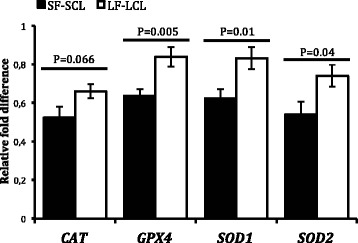
Relative transcript abundance. Values are normalized to the geometric mean of the expression of *GAPDH* and *ACTB* (i.e., reference genes) generated by Genorm software. *CAT*: catalase; *GPx4*: glutathione peroxidase 4; *SOD1*: superoxide dismutase 1; *SOD2*: superoxide dismutase 2. Comparisons between the groups were analyzed by one-way ANOVA and the P-values are shown. Bars represent mean +/− SEM.

The results are summarized in Figure 3. The abundance of transcript of gene that encodes the enzyme glutathione peroxidase (P = 0.005) was lower in the SF-SCL group than in the LF-LCL group. Also, there was a statistical trend to reduced abundance of transcripts for catalase (P = 0.066) in the SF-SCL group.

These results are in agreement with the results found in the activity analysis of these enzymes. However, SOD1, SOD2 and SOD3 are encoded by different genes and have distinct sub-cellular localizations (cytosolic, mitochondrial and extracellular, respectively). In the present study, the abundance of transcripts of the *SOD1* (P = 0.01) and *SOD2* (P = 0.04) genes was lower in the SF-SCL group, in contrast with the results of total activity of superoxide dismutase found in the endometrium. The *SOD3* transcript abundance was not different between the LF-LCL and SF-SCL groups (P > 0.1).

## Discussion

In the present study analyses of uterine washings allowed the detection and quantification of RS, GSH, and SOD activity, demonstrating that the histotroph has regulatory components of the redox system that might be critical during early embryonic development. In contrast, CAT activity was not detected in uterine washings, despite the fact that CAT protein has been reported previously in uterine washings in cattle [33]. Despite the presence of detectable RS, GSH and SOD activities in uterine washings, they were not modulated by the periovulatory sex steroid milieu. It is possible that due to the diluted nature of the uterine fluid, diminute differences in activities between groups were not detectable. Regulation of the histotroph redox environment is important because it can change the uterine environment quality and can impair embryo development. Yoon et al. showed that excessive reactive oxygen species (ROS) reduced the embryo development rate and increased the number of apoptotic cells in embryos cultured *in vitro*, probably due to endoplasmic reticulum stress [34].

**Figure 4:**
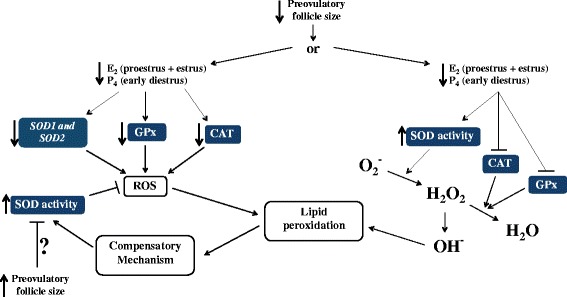
Hypothetical model of ovarian steroid mechanisms regulating the uterine redox environment. Smaller preovulatory follicles produce less estradiol at proestrus/estrus as well as less progesterone during early diestrus, compared to larger follicles. The endocrine milieu associated with smaller preovulatory follicles is characterized by reduced abundance of *SOD1* and *SOD2* transcripts, GPx activity, and CAT activity (left side). Such conditions are prone to increased oxidative stress, resulting in greater lipid peroxidation. Compensatory mechanisms, such as increased total SOD activity, maintain intra-uterine redox homeostasis, as suggested by the similar reactive species concentrations between the two groups. An alternative, integrative explanation (right side) for the results is that the high SOD activity in the presence of reduced CAT and GPx activity could lead to high levels of free hydroxyl radicals that are highly reactive, and could increase lipid peroxidation. We speculate that changes in the mechanisms controlling the redox status in the uterus of cows ovulating smaller follicles are associated with the lower fertility reported for this category of animals.

In the endometrium, there was no difference in the amount of RS between the groups; however, the SF-SCL group presented increased lipid peroxidation and SOD total activity but reduced abundance of *SOD1* and *SOD2* transcripts and no difference in *SOD3.* Additionally, the SF-SCL group exhibited reduced GPx and CAT enzyme activity. It is likely that lower antioxidant activity (i.e., CAT and GPx activities and possibly SOD at an earlier time point) in the SF-SCL group provided an environment that was relatively more prone to lipid peroxidation than that found in the LF-LCL group. One possibility to the relative increase in SOD activity is that the environment with potentially more oxidative potential, as observed in the SF-SCL group, triggered a compensatory mechanism to promote cell survival (Figure 4). In fact, under physiological or low lipid peroxidation environments, cells stimulate their survival through antioxidant defense systems, mounting an adaptive stress response [35]. This adaptive oxidative stress response was also observed in yeast [36,37] and human lymphocytes [38]. An alternative, integrative explanation for the results is that the high SOD activity in the presence of reduced CAT and GPx activity could lead to high levels of free hydroxyl radicals that are highly reactive, and could increase lipid peroxidation, in that case, the higher concentrations of progesterone may be having a inhibitory effect on the SOD activity that is opposite to what has been described in human endometrial cells [39].

For both possibilities above mentioned, the SF-SCL has potentially more oxidative potential, which may be harmful to the quality of uterine environment. In fact, using a bovine endometrial transcriptome study Ponsuksili et al. showed that the NRF2- mediated oxidative stress response was a pathway enriched in the low receptive endometrium at day 7 of the estrous cycle in comparison to the high receptive endometrium [40].

In ruminants little is known about redox status and oxidative stress in the endometrium or uterine environment and their modulation by sex steroids hormones. In sheep, recent study showed that activity of antioxidants enzymes, such as CAT, GPx and SOD2, are up-regulated during pregnancy progress in the endometrium [41]. According to the authors the increase of antioxidants enzymes between day 16 and 21 of pregnancy is a survival response during the transition from the implantation period to the post-implantation period [41] that is important to prevent a possible oxidative insult in early pregnancy [42]. In cattle, some studies associated the oxidative stress with health disorders and immune function of dairy cattle (reviewed by Sordillo & Aitken [43]).

According to studies conducted in other species, there appears to be regulation of the redox mechanisms by sex hormones. It has been demonstrated in pigs that E2 exerts antioxidant activity by inhibiting the H_2_O_2_-induced apoptosis of luteal and follicular cells [44]. Exogenous E2 increased GPx activity in the uterus of rats [17] and reduced the total SOD activity in the uterus of mice [45]. In the uterus of immature rats, E2 increased uterine peroxidase capacity in a dose-dependent manner, and this regulation was both transcription- and translation-dependent [18].

In mice, the E2-mediated reduction in SOD activity appeared to be associated with an increase in the membrane fluidity of endometrial cells [45]. According to the theory of membrane fluidity, which was described by Laloraya [46], during the process of embryo implantation, the increased fluidity of the membranes of endometrial cells, which is caused by a slight increase in lipid peroxidation, aids the fusion of the trophectoderm with the endometrial cells. Interestingly, this increased lipid peroxidation is caused by increased superoxide anion concentrations, which is in turn caused by a decrease in SOD activity. In the present study, the group that received greater exposure to E2 during proestrus/estrus had lower total SOD activity but showed reduced lipid peroxidation, in contrast with the theory of membrane fluidity. An important fact is that the analyses of the present study were performed on Day 7 of the estrous cycle, which was well before the implantation period; however, it is possible that the uterus was in preparation for this event and that preparation was regulated by the periovulatory endocrine milieu.

Little is known about the effects of P4 on the regulation of antioxidant mechanisms. Ohwada et al. showed that P4 did not alter SOD activity in mice [17]. However, in the uterus of rats, P4 appears not only to increase glutathione reductase activity but to also modulate the activity of this enzyme after previous exposure to E2. This process might be a mechanism by which P4 prevents the potentially toxic effects of E2 [19]. It has been demonstrated in sheep that the use of physiological doses of E2 and P4, and a combination of both E2 and P4 reduced the activity of SOD1 in the endometrium [20]. Thus, E2 and P4 work together in the regulation of different important molecules to control the redox environment and tissue and organ environments.

## Conclusions

According to the results of the present study, it could be concluded that cows that ovulated a smaller follicle had decreased redox capacity and consequently increased lipid peroxidation in the endometrium, during the subsequent early diestrus. We speculate that the redox environment found in the group with smaller ovulatory follicles might be one of the causes of the reduced fertility found in these animals, as described in the literature.

## Abbreviations

ACTB: Actin, beta
NADPH: Beta-nicotinamide adenine dinucleotide phosphate reduced
CAT: Catalase
CV: Coefficient of variation
CL: Corpus luteum
U: Enzymatic units
E2: Estradiol
EB: Estradiol benzoate
FU: Fluorescence units
GSH: Glutathione
GPx: Glutathione peroxidase
GAPDH: Glyceraldehyde-3-phosphate dehydrogenase
GnRH: Gonadotropin-releasing hormone
LF-LCL: Large follicle-large CL group
MDA: Malondialdehyde
GSSG: Oxidised glutathione
PPIA: Peptidylprolyl isomerase A (cyclophilin A)
POF: Preovulatory follicle
P4: Progesterone
PGF: Prostaglandin F2α
ROS: Reactive oxygen species
RS: Reactive species
GSH: Reduced glutathione
SF-SCL: Small follicle-small CL group
SOD: Superoxide dismutase
TBA: Thiobarbituric acid
TBARS: Thiobarbituric acid reactive species
